# Replication and cross-validation of type 2 diabetes subtypes based on clinical variables: an IMI-RHAPSODY study

**DOI:** 10.1007/s00125-021-05490-8

**Published:** 2021-06-10

**Authors:** Roderick C. Slieker, Louise A. Donnelly, Hugo Fitipaldi, Gerard A. Bouland, Giuseppe N. Giordano, Mikael Åkerlund, Mathias J. Gerl, Emma Ahlqvist, Ashfaq Ali, Iulian Dragan, Andreas Festa, Michael K. Hansen, Dina Mansour Aly, Min Kim, Dmitry Kuznetsov, Florence Mehl, Christian Klose, Kai Simons, Imre Pavo, Timothy J. Pullen, Tommi Suvitaival, Asger Wretlind, Peter Rossing, Valeriya Lyssenko, Cristina Legido-Quigley, Leif Groop, Bernard Thorens, Paul W. Franks, Mark Ibberson, Guy A. Rutter, Joline W. J. Beulens, Leen M. ‘t Hart, Ewan R. Pearson

**Affiliations:** 1 Department of Epidemiology and Data Science, Amsterdam Public Health Institute, Amsterdam UMC, Location VUMC, Amsterdam, the Netherlands; 2 Department of Cell and Chemical Biology, Leiden University Medical Center, Leiden, the Netherlands; 3 Division of Population Health & Genomics, School of Medicine, University of Dundee, Dundee, UK; 4 Genetic and Molecular Epidemiology Unit, Department of Clinical Sciences, CRC, Lund University Diabetes Centre, Lund University, Malmö, Sweden; 5 Lipotype GmbH, Dresden, Germany; 6 Steno Diabetes Center Copenhagen, Gentofte, Denmark; 7 Vital-IT Group, SIB Swiss Institute of Bioinformatics, Lausanne, Switzerland; 8 Eli Lilly Regional Operations GmbH, Vienna, Austria; 9 1st Medical Department, LK Stockerau, Niederösterreich, Austria; 10 Cardiovascular and Metabolic Disease Research, Janssen Research & Development, Spring House, PA USA; 11 Institute of Pharmaceutical Science, Faculty of Life Sciences and Medicines, King’s College London, London, UK; 12 Department of Diabetes, Guy’s Campus King’s College London, London, UK; 13 Section of Cell Biology and Functional Genomics, Division of Diabetes, Endocrinology and Metabolism, Department of Metabolism, Digestion and Reproduction, Imperial College London, London, UK; 14 Department of Clinical Science, Center for Diabetes Research, University of Bergen, Bergen, Norway; 15 Genomics, Diabetes and Endocrinology Unit, Department of Clinical Sciences Malmö, Lund University Diabetes Centre, Skåne University Hospital, Malmö, Sweden; 16 Finnish Institute of Molecular Medicine, Helsinki University, Helsinki, Finland; 17 Center for Integrative Genomics, University of Lausanne, Lausanne, Switzerland; 18 Department of Nutrition, Harvard School of Public Health, Boston, MA USA; 19 Lee Kong Chian School of Medicine, Nanyang Technological University, Singapore, Republic of Singapore; 20 Julius Center for Health Sciences and Primary Care, University Medical Center Utrecht, Utrecht, the Netherlands; 21 Department of Biomedical Data Sciences, Section of Molecular Epidemiology, Leiden University Medical Center, Leiden, the Netherlands

**Keywords:** Clusters, C-peptide, Cross-validation, HDL-cholesterol, Type 2 diabetes

## Abstract

**Aims/hypothesis:**

Five clusters based on clinical characteristics have been suggested as diabetes subtypes: one autoimmune and four subtypes of type 2 diabetes. In the current study we replicate and cross-validate these type 2 diabetes clusters in three large cohorts using variables readily measured in the clinic.

**Methods:**

In three independent cohorts, in total 15,940 individuals were clustered based on age, BMI, HbA_1c_, random or fasting C-peptide, and HDL-cholesterol. Clusters were cross-validated against the original clusters based on HOMA measures. In addition, between cohorts, clusters were cross-validated by re-assigning people based on each cohort’s cluster centres. Finally, we compared the time to insulin requirement for each cluster.

**Results:**

Five distinct type 2 diabetes clusters were identified and mapped back to the original four All New Diabetics in Scania (ANDIS) clusters. Using C-peptide and HDL-cholesterol instead of HOMA2-B and HOMA2-IR, three of the clusters mapped with high sensitivity (80.6–90.7%) to the previously identified severe insulin-deficient diabetes (SIDD), severe insulin-resistant diabetes (SIRD) and mild obesity-related diabetes (MOD) clusters. The previously described ANDIS mild age-related diabetes (MARD) cluster could be mapped to the two milder groups in our study: one characterised by high HDL-cholesterol (mild diabetes with high HDL-cholesterol [MDH] cluster), and the other not having any extreme characteristic (mild diabetes [MD]). When these two milder groups were combined, they mapped well to the previously labelled MARD cluster (sensitivity 79.1%). In the cross-validation between cohorts, particularly the SIDD and MDH clusters cross-validated well, with sensitivities ranging from 73.3% to 97.1%. SIRD and MD showed a lower sensitivity, ranging from 36.1% to 92.3%, where individuals shifted from SIRD to MD and vice versa. People belonging to the SIDD cluster showed the fastest progression towards insulin requirement, while the MDH cluster showed the slowest progression.

**Conclusions/interpretation:**

Clusters based on C-peptide instead of HOMA2 measures resemble those based on HOMA2 measures, especially for SIDD, SIRD and MOD. By adding HDL-cholesterol, the MARD cluster based upon HOMA2 measures resulted in the current clustering into two clusters, with one cluster having high HDL levels. Cross-validation between cohorts showed generally a good resemblance between cohorts. Together, our results show that the clustering based on clinical variables readily measured in the clinic (age, HbA_1c_, HDL-cholesterol, BMI and C-peptide) results in informative clusters that are representative of the original ANDIS clusters and stable across cohorts. Adding HDL-cholesterol to the clustering resulted in the identification of a cluster with very slow glycaemic deterioration.

**Graphical abstract:**

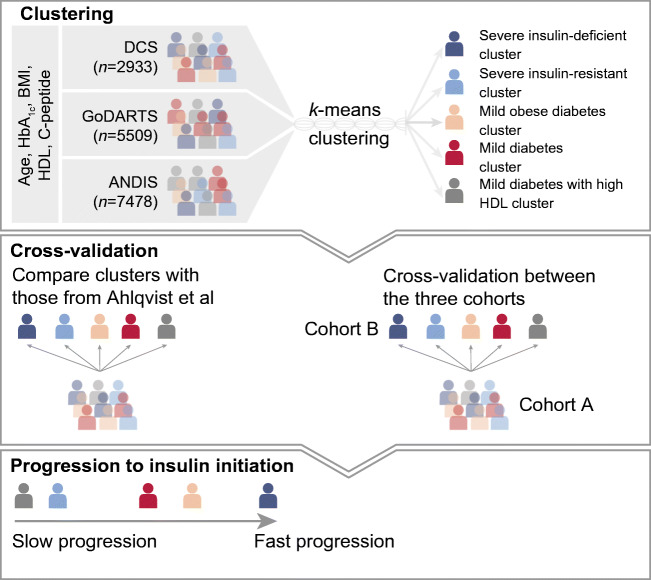

**Supplementary Information:**

The online version contains peer-reviewed but unedited supplementary material available at 10.1007/s00125-021-05490-8.

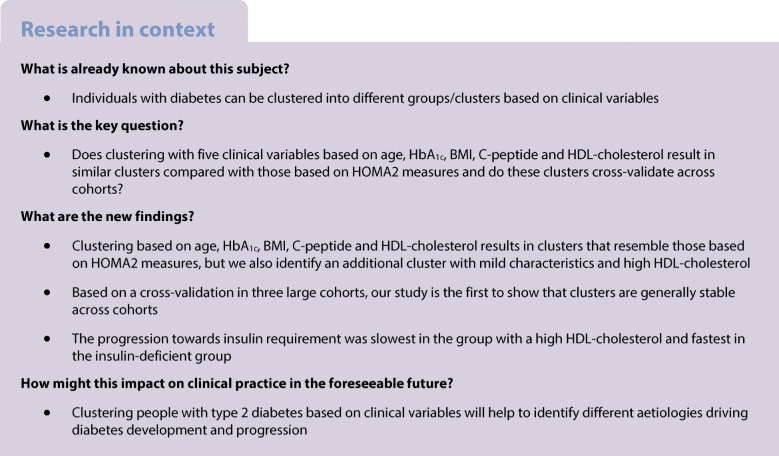



## Introduction

A recent study stratified people with any form of diabetes into five clusters based on six clinical variables, i.e. age, GAD antibodies, BMI, HbA_1c_, insulin resistance (HOMA2-IR) and beta cell function estimates (HOMA2-B) [1]. The five clusters were characterised by autoimmunity (severe autoimmune diabetes [SAID]), insulin deficiency (severe insulin-deficient diabetes [SIDD]), insulin resistance (severe insulin-resistant diabetes [SIRD]), high BMI (mild obesity-related diabetes [MOD]) and the last without any extreme characteristics other than high age (mild age-related diabetes [MARD]) [1]. Clustering of people with diabetes has been repeated successfully in several other studies based on these variables in people of European descent and of other ethnicities and based on different clinical measures [2–9]. In addition, the original and subsequent papers have shown that people in different clusters have different risks for a number of diabetes-related outcomes [1–4]. The autoimmunity and insulin-deficient clusters were defined by high HbA_1c_ at diagnosis, had higher risk for ketoacidosis and retinopathy [2, 7], and progressed more rapidly onto insulin relative to the other clusters [1]. Moreover, a recent study comprising multiple cohorts enriched for cardiovascular risk assigned people to the clusters identified by Ahlqvist et al [1] based on the distance to the respective cluster centres. In this study, people in the SIDD cluster showed higher risk of major adverse cardiovascular events [5]. For the insulin-resistant cluster, a higher frequency of non-alcoholic fatty liver disease has been observed and people in this group were at increased risk of developing chronic kidney disease [1]. As HOMA2 calculations require fasting insulin or C-peptide and fasting glucose, their measurement is not routine in clinical practice.

The aim of the current study is to perform a systematic replication and cross-validation of clustering based on five routine clinical variables in three large international cohorts (Diabetes Care System [DCS], All New Diabetics in Scania [ANDIS], Genetics of Diabetes Audit and Research Tayside Study [GoDARTS]). In ANDIS, we directly compare the current clusters with those identified in the original study [1].

## Methods

### Cohort descriptions

Data from 15,940 individuals with type 2 diabetes from three cohorts, DCS (Netherlands), GoDARTS (Scotland) and ANDIS (Sweden), were used in this cross-sectional study within the RHAPSODY consortium. RHAPSODY (Risk Assessment and ProgreSsiOn of Diabetes, https://imi-rhapsody.eu) is an Innovative Medicine Initiative project and one of the aims is to improve the segmentation of people with type 2 diabetes, supporting the implementation of novel strategies for diabetes prevention and treatment. Inclusion criteria for RHAPSODY were age of diagnosis ≥35, clinical data available within 2 years after diagnosis, GAD negative, no missing data in one of the five clinical measures used for clustering and the presence of genome-wide association study (GWAS) data.

#### Hoorn DCS cohort

The Hoorn DCS cohort is an open prospective cohort started in 1998 with currently over 14,000 individuals with type 2 diabetes from the north-west part of the Netherlands [10]. The study has been approved by the Ethical Review Committee of the Vrije Universiteit University Medical Center, Amsterdam. People visit DCS annually to monitor their diabetes. During this visit, multiple measurements are collected as part of routine care, including anthropometric and laboratory measurements. Measurements were used anonymously. Individuals were informed about the use of their data and were offered an opt-out. All laboratory measurements were done on samples taken in a fasted state. HbA_1c_ measurements were performed using the turbidimetric inhibition immunoassay for haemolysed whole EDTA blood (Cobas c501, Roche Diagnostics, Mannheim, Germany, run CV 1.6%) [10]. HDL-cholesterol (mmol/l) was measured enzymatically (Cobas c501, Roche Diagnostics). C-peptide was measured on a DiaSorin Liaison (DiaSorin, Saluggia, Italy). In total, 2953 individuals matched the inclusion criteria.

#### GoDARTS

For clinical purposes, individuals with diabetes mellitus from the Tayside region of Scotland (*n* = 391,274; January 1996) were added to the Diabetes Audit and Research Tayside Study (DARTS) register [11]. Retrospective and prospective longitudinal anonymised data were collected, including data on prescribing and biochemistry and clinical data. All laboratory measurements were measured in a non-fasted state. People with type 2 diabetes were asked to participate in the Genetics of DARTS study (GoDARTS), which currently includes over 10,000 individuals with type 2 diabetes [11]. The GoDARTS study was approved by the Tayside Medical Ethics Committee. Informed consent was obtained from all participants. C-peptide was measured on a DiaSorin Liaison. In total, 5509 individuals matched the inclusion criteria.

#### ANDIS

The ANDIS cohort aims to recruit all people with incident diabetes within Scania County, Sweden. Recruitment started in January 2008 until November 2016. People are included in the study close to diagnosis, with a median of 40 days (IQR 12–99). All laboratory measurements were measured in a fasted state. HbA_1c_ measurements were obtained from the Clinical Chemistry database. C-peptide was determined with an electro-chemiluminescence immunoassay on a Cobas e411 (Roche Diagnostics) or by a radioimmunoassay (Human C-peptide radioimmunoassay; Linco, St Charles, MO, USA; or Peninsula Laboratories, Belmont, CA, USA). In total, 7478 individuals matched the inclusion criteria.

### Statistical analysis

Clustering was performed on five risk factors for type 2 diabetes progression [12]: age at first visit (years); BMI (kg/m^2^); HbA_1c_ (mmol/mol); HDL-cholesterol (mmol/l); and C-peptide (nmol/l). C-peptide was included as a proxy of insulin resistance and, to some extent, beta cell function (electronic supplementary material [ESM] Table 1) in absence of fasting glucose in GoDARTS (preventing the use of HOMA). HDL-cholesterol levels were included as lower HDL-cholesterol has previously been recognised as a risk factor for time to insulin requirement [12]. Clustering was performed separately in each cohort and stratified by sex. Clusters were defined based on *k*-means using the *kmeansruns* function in the R package *fpc* (https://cran.r-project.org/web/packages/fpc/index.html)*.* The optimal number of clusters was determined using the gap statistic across the three cohorts [13], this being defined as the point where the curve of the gap statistic vs the number of clusters flattened, with little added value of increasing the number of clusters. The stability of the clusters was assessed in two ways. The clusters identified here in ANDIS using C-peptide instead of HOMA2 were compared with their previously published clusters based on HOMA2 [1]. Second, identified clusters were cross-validated between cohorts to assess their stability. For this, individuals from cohort A were assigned to clusters based on the cluster centres of each of the clusters identified in cohort B. This approach will quantify the probability that an individual in cohort A will be assigned to the same cluster, but based on the clustering model for cohort B. Next, predicted clusters in cohort A based on the clusters of cohort B were compared with the ‘real’ clusters of cohort A. This was done for each of the three pairwise comparisons (DCS–GoDARTS, DCS–ANDIS, GoDARTS–ANDIS). Agreement between clusters was assessed based on the specificity and sensitivity.

Time to insulin requirement was defined as the period until an individual started sustained (more than 6 months in duration) insulin treatment or required insulin, defined as ≥2 HbA_1c_ measurements >69 mmol/mol (8.5%) at least 3 months apart and when on ≥2 non-insulin glucose-lowering drugs. Cox proportional hazard models were used where one cluster was tested against the other clusters as a reference group in each individual cohort. Thereafter, results were meta-analysed using random effects meta-analysis using the *metagen* function from the *meta* package (https://cran.r-project.org/web/packages/meta/index.html). Analyses were performed using R statistics (version 3.6.2; https://www.r-project.org/). Figures were produced using the R packages *ggplot2* (v3.3.0) (https://cran.r-project.org/web/packages/ggplot2/index.html) and *omicCircos* (v1.22.0) (http://www.bioconductor.org/packages/release/bioc/html/OmicCircos.html).

## Results

### Clustering in three large cohorts based on clinical measures

**Fig. 1:**
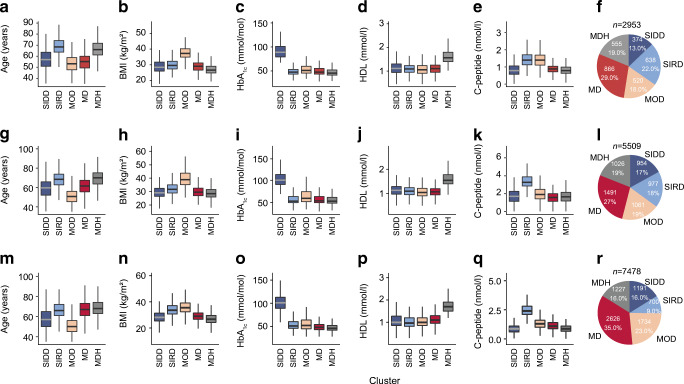
Characteristics of the clusters. (**a**–**e**, **g**–**k**, **m**–**q**) Characteristics of the five clusters across the three cohorts; DCS (**a**–**e**), GoDARTS (**g**–**k**) and ANDIS (**m**–**q**); *x*-axis, cluster; *y*-axis, age, BMI, HbA_1c_, HDL-cholesterol and C-peptide. (**f**, **l**, **r**) Frequency and percentage of individuals in each of the clusters; DCS (**f**), GoDARTS (**l**) and ANDIS (**r**)

In this cross-sectional study, 15,940 individuals from three cohorts were included, for which baseline characteristics are given in Table 1. The characteristics of the three cohorts were generally comparable, with the majority male participants and an average age of around 60 years. Individuals were clustered based on age, BMI, HbA_1c_, C-peptide and HDL-cholesterol. The optimal number of clusters was based on the gap statistic across the three cohorts. In GoDARTS the optimal number of clusters was five, with lower gap statistics from six onwards. In DCS and ANDIS, the increase in gap statistic showed a clear stabilisation after five clusters. Therefore, we considered five the most optimal number of clusters (ESM Fig. 1a). The first cluster comprised 13–17% of the individuals included. It was characterised by high HbA_1c_, but, compared with the other clusters, participants were younger with lower BMI, C-peptide and HDL-cholesterol levels. When compared with the original clusters in ANDIS [1], this cluster was most similar to the SIDD cluster with a sensitivity (SEM) of 90.7% (CI 88.4%, 92.6%; Fig. 1, ESM Fig. 1b) [1]. Between 9% and 22% of individuals clustered to a cluster with high C-peptide levels and age, but relatively lower HbA_1c_ and HDL-cholesterol levels, suggestive of insulin resistance. Indeed, compared with the ANDIS clusters, this cluster resembled most the SIRD cluster with an SEM of 92.4% (CI 89.7%, 94.6%; Fig. 1, ESM Fig. 1b) [1]. The third cluster comprised participants with high BMI and the youngest age and relatively lower levels of HbA_1c_ and HDL-cholesterol. It was most similar to the originally described MOD cluster with an SEM of 80.6% (CI 78.4%, 82.7%) and comprised 18–23% of the individuals included in the study. The fourth and fifth clusters were most similar to the MARD cluster and showed a combined sensitivity of 79.1% (CI 77.5%, 80.6%) against the MARD cluster in ANDIS (Fig. 1, ESM Fig. 1b) [1]. The fourth cluster, which was also the largest, encompassing 29–35% of the individuals, showed no extreme characteristics and was termed mild diabetes (MD). The fifth cluster was characterised by higher age and HDL-cholesterol and was termed mild diabetes with high HDL-cholesterol (MDH), and comprised 16–19% of the individuals (Fig. 1). Between male and female participants there were small differences in characteristics, but the overall differences between clusters were similar across both sexes (ESM Fig. 2).

**Table 1:** Characteristics of the included individuals of the three cohorts

Variable	DCS	GoDARTS	ANDIS
*n*	2953	5509	7478
Male, %	55.9	56.3	60.1
Age, years	60.2 (53.1–66.9)	62.5 (54.5–70.0)	62.0 (54.0–69.8)
BMI, kg/m^2^	29.5 (26.7–33.2)	31.0 (27.6–35.1)	30.8 (26.9–34.0)
HbA_1c_, mmol/mol	49.7 (44.0–60.7)	58.0 (50.0–79.0)	62.3 (45.0–74.0)
HbA_1c_, %	6.7 (6.2–7.7)	7.5 (6.7–9.4)	7.9 (6.3–8.9)
C-peptide, nmol/l	1.0 (0.8–1.4)	1.9 (1.3–2.6)	1.3 (0.9–1.5)
HDL-cholesterol, mmol/l	1.2 (0.97–1.37)	1.1 (1.0–1.4)	1.2 (0.9–1.4)
LDL-cholesterol, mmol/l	3.0 (2.3–3.7)	2.7 (2.0–3.4)	3.2 (2.5–3.9)
Triacylglycerol, mmol/l	1.7 (1.2–2.3)	2.3 (1.6–3.3)	2.2 (1.2–2.4)
Glucose-lowering medication, %	61.5	19.0	59.6

Data are displayed as median (IQR), except where indicated otherwise

### Clusters cross-validate between the three cohorts

To assess the stability across cohorts, clusters were cross-validated between cohorts. Clusters generally cross-validated well between the three cohorts (ESM Fig. 3, ESM Table 2). The SIDD and MDH clusters showed the highest sensitivity of the five clusters identified, ranging from 85.6% (CI 83.5%, 87.6%) to 97.1% (CI 94.8%, 98.5%) in SIDD and from 73.3% (CI 69.5%, 77.0%) to 92.9% (CI 91.3%, 94.3%) in MDH (ESM Fig. 3, ESM Table 2). The SIRD and MD clusters performed generally worst in terms of sensitivity, with sensitivities ranging from 36.1% (CI 32.3%, 39.9%) to 92.3% (CI 90.1%, 94.2%) in SIRD and from 40.8% (CI 38.9%, 42.7%) to 78.1% (CI 75.9%, 80.2%) in MD. Individuals clustered to SIRD were classified as MD and vice versa (ESM Fig. 3, ESM Table 2). The sensitivity of the MOD cluster ranged from 55.0% (CI 52.6%, 57.3%) to 93.2% (CI 91.5%, 94.7%).

### Clusters are different in their progression to insulin requirement

Next, we assessed differences between clusters in terms of progression towards insulin initiation or requirement. As expected, the SIDD cluster showed the fastest progression (HR 3.40 [CI 1.72, 6.72]) compared with the other clusters (Table 2, ESM Fig. 4). The SIRD group showed slower progression (0.59 [0.46, 0.76]). The clusters MD and MDH also showed differences in their progression, where MDH showed the slowest progression compared with the other clusters (0.44 [0.33, 0.59]), also slower than MD (0.81 [0.63, 1.06]).

**Table 2:** Meta-analysis results for time to insulin requirement

Cluster	HR (95%CI)	*p* value
SIDD	3.40 (1.72, 6.72)	4.24 × 10^−4^
SIRD	0.59 (0.46, 0.76)	4.15 × 10^−5^
MOD	1.17 (0.64, 2.12)	0.61
MD	0.81 (0.63, 1.06)	0.12
MDH	0.44 (0.33, 0.59)	3.84 × 10^−8^

Each cluster was tested against the four other clusters as reference group

## Discussion

Based on five clinical variables, people with type 2 diabetes from three large European cohorts were assigned to five separate clusters. Clusters were successfully cross-validated against the clustering reported by Ahlqvist et al [1] but also between cohorts included.

Even though we used slightly different variables for clustering, i.e. C-peptide and HDL-cholesterol instead of HOMA2 measures [1], people were clustered largely to the same clusters in a direct comparison with previously published clusters in ANDIS. The insulin-deficient cluster (SIDD) was defined by a high HbA_1c_, the insulin-resistant cluster (SIRD) by a high C-peptide and the obese cluster (MOD) by a high BMI. The previously identified MARD cluster [1] could be further divided into two clusters of people with a low (MD cluster) and a high HDL-cholesterol (MDH cluster). Including HDL-cholesterol resulted in two clusters with mild characteristics, where one had high HDL-cholesterol and one cluster had generally a low HDL-cholesterol. A subset of the SIRD cluster was classified as MD, which is most likely due to the use of C-peptide and HDL-cholesterol instead of HOMA2 measures.

In addition to a comparison with the original ANDIS clusters, in the current study we also cross-validated the clusters across cohorts. Clusters cross-validated generally well and the best sensitivity was observed in the SIDD and MDH clusters. For SIRD and MD a lower sensitivity was observed. Individuals that were classified in one cohort to SIRD or MOD were classified as MD in a second cohort and vice versa. The characteristics of particularly SIRD and MD are very similar, with the sole difference being higher levels of C-peptide in the SIRD cluster. This could explain the difference in classification in the two cohorts.

A limitation of the current study is that individuals in DCS and GoDARTS were not clustered based on clinical data collected at the time of diagnosis prior to treatment. Different treatment regimens could have had an influence on the clustering. However, it should be noted that ANDIS was clustered based on data collected at the time of diagnosis and in GoDARTS a smaller group was treated at baseline compared with DCS. Therefore, treatment effects did not seem to have a major influence on the clustering or the cross-validation.

The progression towards insulin requirement of the identified clusters resembled that of the original clusters in ANDIS [1]. The SIDD group showed the fastest progression, followed by MOD. The SIRD group showed a generally slower progression in our study. The MDH cluster that we additionally identified showed the slowest progression of all clusters. This shows that adding HDL-cholesterol to the clustering allows the identification of a separate group among those with mild diabetes with very low risk of glycaemic deterioration towards insulin requirement.

### Conclusion

In the current study, clusters were identified in three cohorts, based on five different clinical characteristics. We show that clusters based on random or fasted C-peptide instead of HOMA2 measures resemble those based on HOMA2 measures. By adding HDL-cholesterol, we identified one additional cluster with mild characteristics. Cross-validation between cohorts showed that there was generally a good resemblance between cohorts. Together, our results show that the clustering is generally stable across cohorts, and also when the clustering includes C-peptide instead of HOMA measures. The novel MDH cluster represents a group of people with mild diabetes and very low risk of glycaemic deterioration towards insulin requirement.

## Supplementary information

ESM (PDF 829 kb)

## Data Availability

Steering committees of the individual cohorts will consider reasonable requests for sharing of de-identified patient-level data.

## Abbreviations

ANDIS: All New Diabetics in Scania
DCS: Diabetes Care System
GoDARTS: Genetics of Diabetes Audit and Research Tayside Study
MARD: Mild age-related diabetes
MD: Mild diabetes
MDH: Mild diabetes with high HDL-cholesterol
MOD: Mild obesity-related diabetes
RHAPSODY: Risk Assessment and ProgreSsiOn of DIabetes
SIDD: Severe insulin-deficient diabetes
SIRD: Severe insulin-resistant diabetes
