# Supplementary material for: Replication and cross-validation of type 2 diabetes subtypes based on clinical variables: an IMI-RHAPSODY study
Source: Diabetologia. 2021 Jun 10;64(9):1982–9. doi: 10.1007/s00125-021-05490-8 (PMC8382625; doi:10.1007/s00125-021-05490-8)
Supplement: Supplementary file 1 — (PDF 829 kb) [file 125_2021_5490_MOESM1_ESM.pdf]

ESM Tables

ESM Table 1 Correlation between HOMA2-B, HOMA2-IR and C-peptide, HDL-cholesterol in DCS and ANDIS.

**HOMA measure**	**Var**	**Cohort**	**ρ**
HOMA2-IR	C-peptide	DCS	0.98
		ANDIS	0.93
HOMA2-B	C-peptide	DCS	0.63
		ANDIS	0.62
HOMA2-IR	HDL-cholesterol	DCS	-0.22
		ANDIS	-0.27
HOMA2-B	HDL-cholesterol	DCS	-0.10
		ANDIS	-0.05

Correlation coefficient based on spearman's Rho.

ESM Table 2 Sensitivity and specificity of the cross-validation of clusters in other studies.

Group	Sensitivity	Specificity	Cohort	Centres
1/SIDD	96.22[94.98-97.23]	98.62[98.3-98.89]	GoDARTS	ANDIS
1/SIDD	97.06[94.8-98.52]	97.32[96.63-97.91]	ANDIS	DCS
1/SIDD	85.64[83.52-87.59]	99.63[99.45-99.77]	DCS	ANDIS
1/SIDD	88.78[86.61-90.72]	98.59[98.21-98.92]	ANDIS	GoDARTS
1/SIDD	77.57[74.79-80.18]	99.19[98.88-99.43]	DCS	GoDARTS
1/SIDD	97.06[94.8-98.52]	96.36[95.56-97.04]	GoDARTS	DCS
2/SIRD	92.29[90.05-94.15]	92[91.33-92.64]	GoDARTS	ANDIS
2/SIRD	36.05[32.32-39.91]	94.43[93.41-95.33]	ANDIS	DCS
2/SIRD	68.57[64.99-72]	82.75[81.83-83.65]	DCS	ANDIS
2/SIRD	56.19[53.02-59.33]	99.29[99-99.52]	ANDIS	GoDARTS
2/SIRD	83.42[80.94-85.7]	89.67[88.75-90.54]	DCS	GoDARTS
2/SIRD	64.26[60.41-67.99]	94.25[93.23-95.17]	GoDARTS	DCS
3/MOD	76.01[73.93-78]	98.45[98.1-98.75]	GoDARTS	ANDIS
3/MOD	73.85[69.84-77.57]	85.16[83.69-86.55]	ANDIS	DCS
3/MOD	54.96[52.58-57.32]	95.73[95.18-96.24]	DCS	ANDIS
3/MOD	93.21[91.53-94.65]	92.72[91.91-93.46]	ANDIS	GoDARTS
3/MOD	62.87[59.88-65.78]	96.27[95.67-96.81]	DCS	GoDARTS
3/MOD	74.23[70.24-77.94]	91.62[90.44-92.69]	GoDARTS	DCS
4/MD	66.15[64.3-67.96]	91.53[90.71-92.3]	GoDARTS	ANDIS
4/MD	51.04[47.65-54.42]	74.46[72.53-76.32]	ANDIS	DCS
4/MD	40.82[38.93-42.73]	81.04[79.91-82.13]	DCS	ANDIS
4/MD	78.14[75.95-80.21]	83.13[81.93-84.27]	ANDIS	GoDARTS
4/MD	67.81[65.37-70.17]	86.96[85.88-87.99]	DCS	GoDARTS
4/MD	66.74[63.5-69.88]	87.83[86.35-89.2]	GoDARTS	DCS
5/MDH	92.75[91.15-94.13]	94.18[93.57-94.74]	GoDARTS	ANDIS
5/MDH	73.33[69.45-76.97]	98.54[97.98-98.98]	ANDIS	DCS
5/MDH	92.91[91.33-94.28]	92.71[92.03-93.34]	DCS	ANDIS
5/MDH	76.02[73.29-78.61]	98.19[97.76-98.56]	ANDIS	GoDARTS
5/MDH	89.47[87.43-91.28]	96.36[95.77-96.89]	DCS	GoDARTS
5/MDH	85.41[82.19-88.24]	97.62[96.93-98.19]	GoDARTS	DCS

SIDD, Severe Insulin-Deficient Diabetes; SIRD, Severe Insulin-Resistant Diabetes cluster; MOD, Mild Obesity-related Diabetes; MD, Mild diabetes; MDH, Mild diabetes with high HDL-cholesterol.

ESM Figures

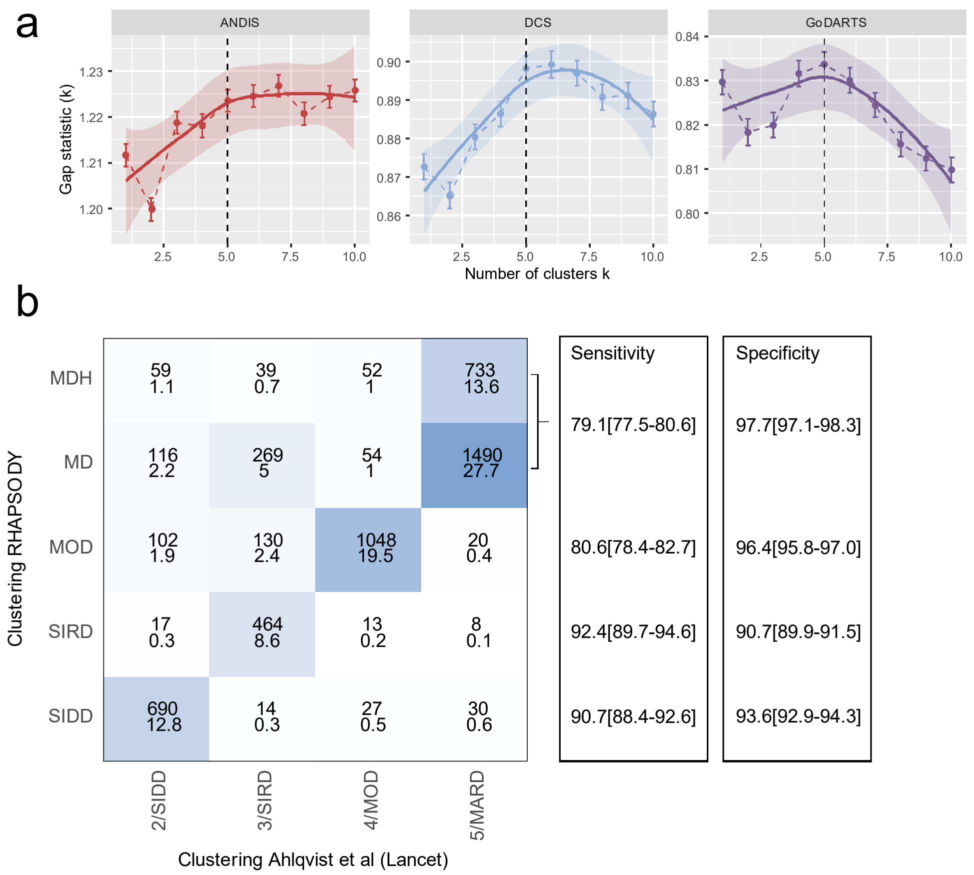

ESM Figure 1 GAP statistic and comparison to clusters based on HOMA2 measures a. Number of clusters versus the GAP statistic in DCS, GoDARTS and ANDIS. The most optimal number of clusters was at five clusters indicated by the dotted line. **b.** Comparison of clustering in ANDIS in the current study versus those identified in Ahlqvist et al.[1]. Numbers on the diagonal represent the absolute overlap (upper number) and the percentage (lower number). In the right panel, the sensitivity and specificity are given with 95% confidence intervals. SIDD, Severe Insulin-Deficient Diabetes; SIRD, Severe Insulin-Resistant Diabetes cluster; MOD, Mild Obesity-related Diabetes; MD, Mild diabetes; MDH, Mild diabetes with high HDL-cholesterol.

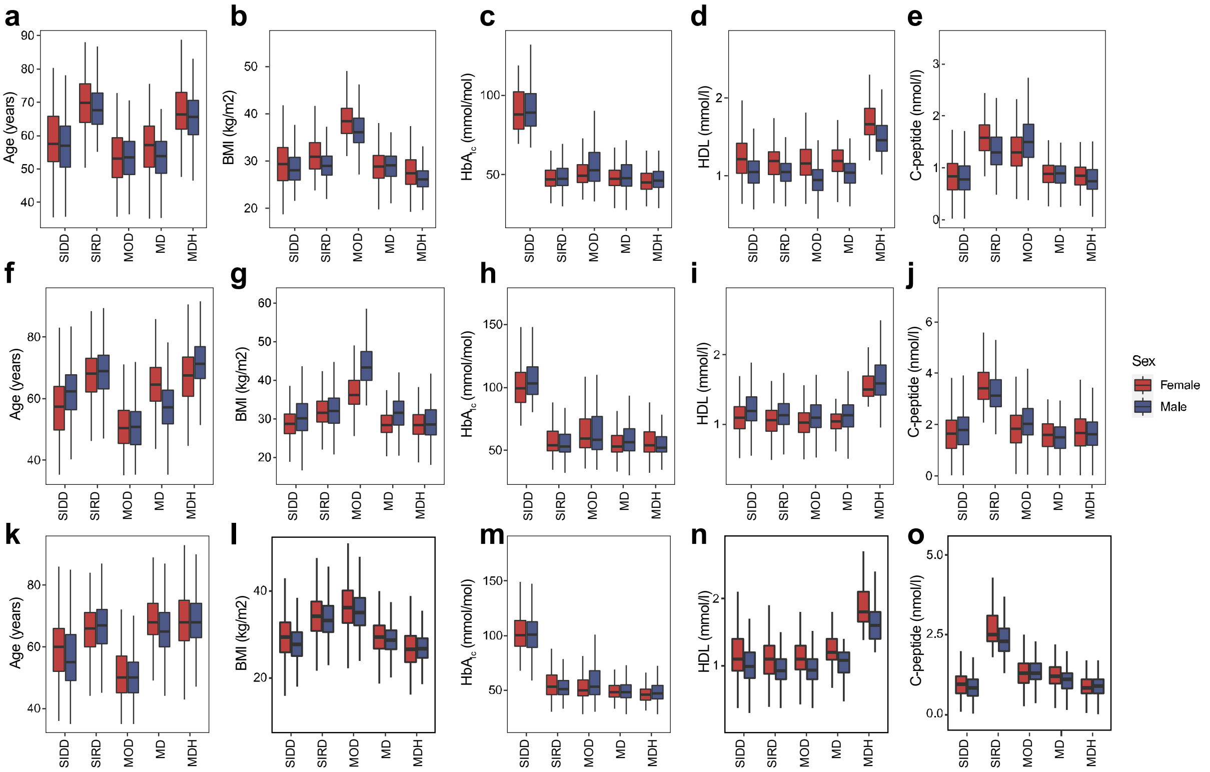

ESM Figure 2 Characteristics of the clusters for males and females separate. a. Characteristics of the five clusters across three cohorts DCS **(a-e)**, GoDARTS **(f-j)** and ANDIS **(k-o)**. X-axis, cluster. Y-axis, age **(a, f, k)**, BMI **(b, g, i)**, HbA1c **(c, h, m)**, HDL-cholesterol **(d, i, n)**, and C-peptide **(e, j, o)**. SIDD, Severe Insulin-Deficient Diabetes; SIRD, Severe Insulin-Resistant Diabetes cluster; MOD, Mild Obesity-related Diabetes; MD, Mild diabetes; MDH, Mild diabetes with high HDL-cholesterol. Red females, blue males.

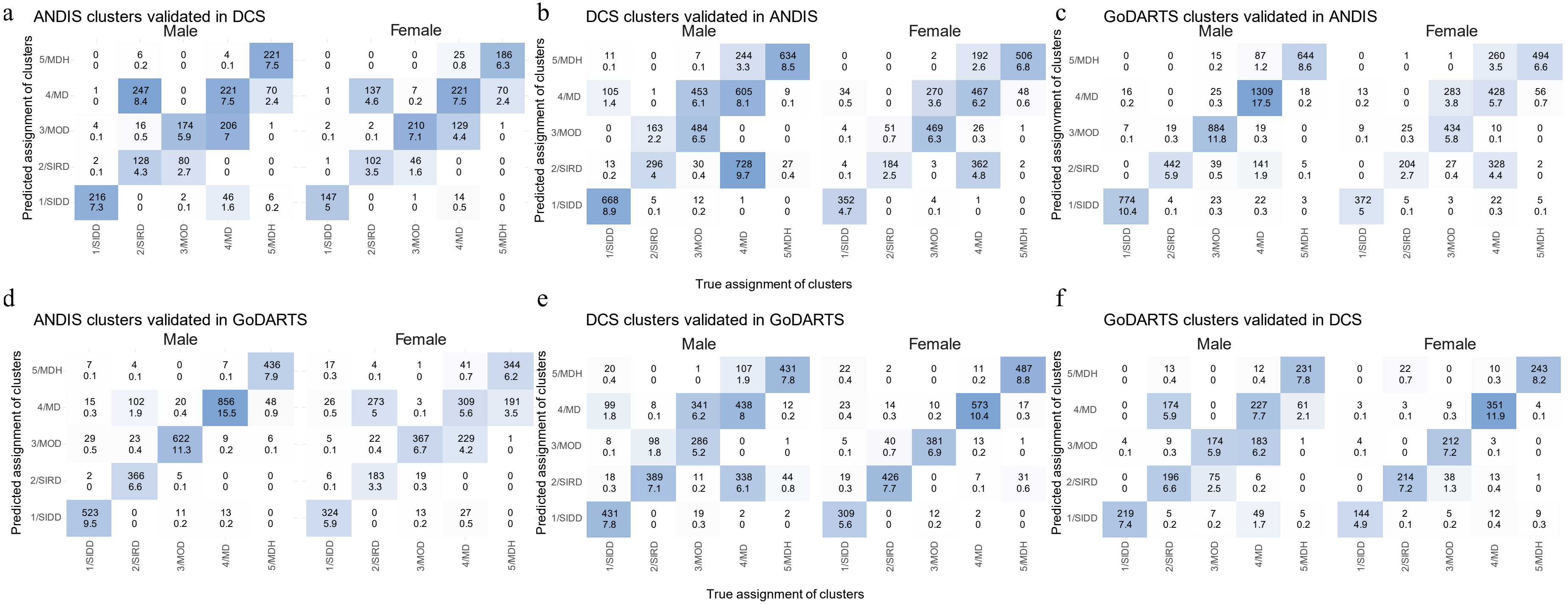

ESM Figure 3 All pairwise comparisons of identified clusters versus predicted clusters. Top number in each cell represents the absolute overlap, the bottom number the percentage of individuals. Individuals were assigned to a cluster in cohort A based on the cluster centres of cohort B and compared to the clusters identified in the cohort A. This resulted in the following combinations: **a** clusters identified in DCS based on ANDIS centres **b** clusters identified in ANDIS based on DCS centres **c** clusters identified in ANDIS based on GoDARTS centres **d** clusters identified in GoDARTS based on ANDIS centres **e** clusters identified in GoDARTS based on DCS centres **f** clusters identified in DCS based on GoDARTS centres. SIDD, Severe Insulin-Deficient Diabetes; SIRD, Severe Insulin-Resistant Diabetes cluster; MOD, Mild Obesity-related Diabetes; MD, Mild diabetes; MDH, Mild diabetes with high HDL-cholesterol.

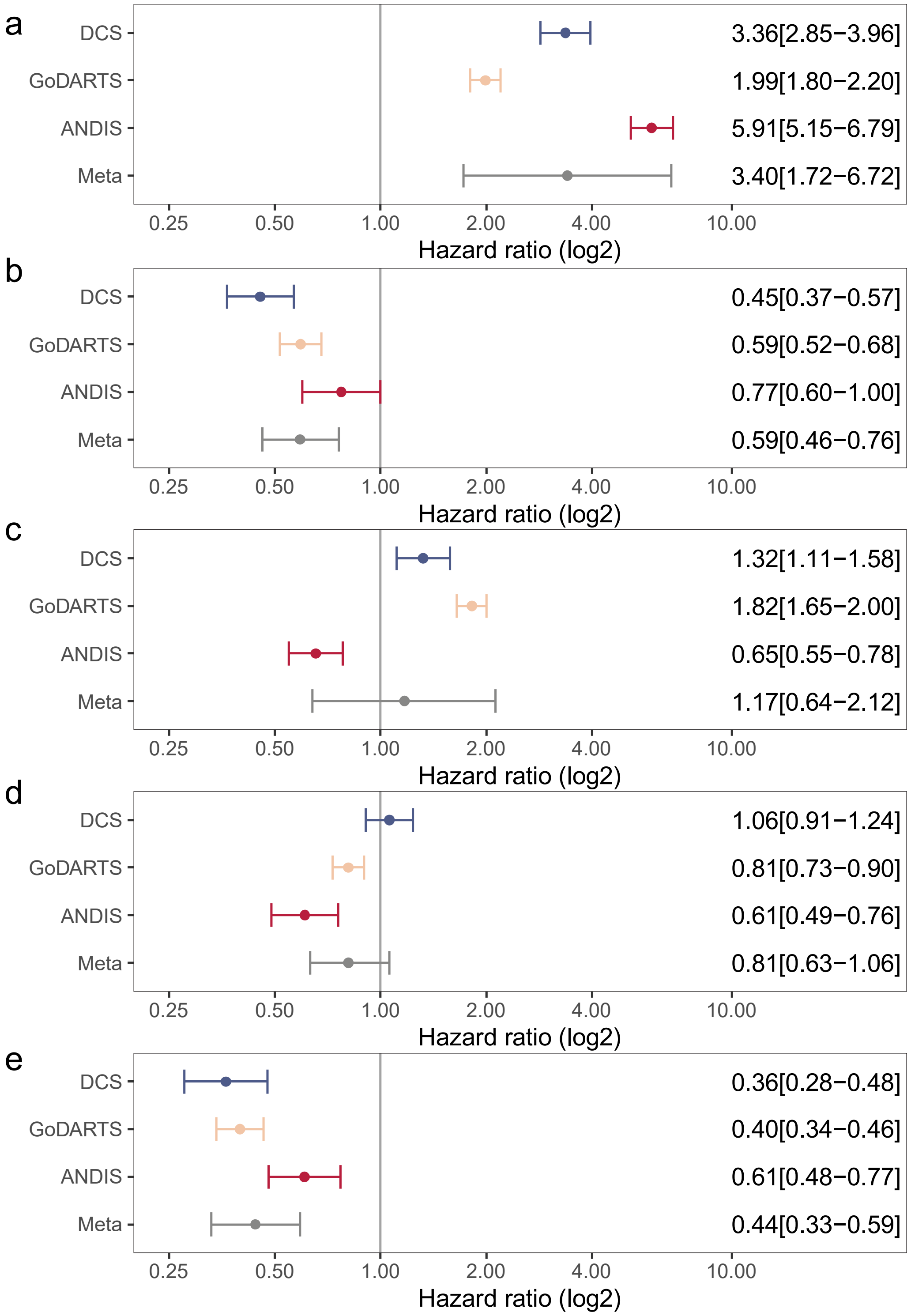

ESM Figure 4 Hazard for time to insulin requirement across clusters. a SIDD, **b** SIRD, **c** MOD, **d** MD, **e** MDH. X-axis, hazard ratio, y-axis cluster. Numbers in panels represent hazards with 95% confidence intervals.
